# Synergistic Inhibition of Protein Fibrillation by Proline and Sorbitol: Biophysical Investigations

**DOI:** 10.1371/journal.pone.0166487

**Published:** 2016-11-21

**Authors:** Sinjan Choudhary, Shreyada N. Save, Nand Kishore, Ramakrishna V. Hosur

**Affiliations:** 1 UM-DAE Centre for Excellence in Basic Sciences, University of Mumbai, Kalina Campus, Mumbai, India; 2 Department of Chemistry, Indian Institute of Technology-Bombay, Mumbai, India; 3 Department of Chemical Sciences, Tata Institute of Fundamental Research, Mumbai, India; Russian Academy of Medical Sciences, RUSSIAN FEDERATION

## Abstract

We report here interesting synergistic effects of proline and sorbitol, two well-known chemical chaperones, in the inhibition of fibrillation of two proteins, insulin and lysozyme. A combination of many biophysical techniques has been used to understand the structural morphology and modes of interaction of the chaperones with the proteins during fibrillation. Both the chaperones establish stronger polar interactions in the elongation and saturation stages of fibrillation compared to that in the native stage. However, when presented as a mixture, we also see contribution of hydrophobic interactions. Thus, a co-operative adjustment of polar and hydrophobic interactions between the chaperones and the protein surface seems to drive the synergistic effects in the fibrillation process. In insulin, this synergy is quantitatively similar in all the stages of the fibrillation process. These observations would have significant implications for understanding protein folding concepts, in general, and for designing combination therapies against protein fibrillation, in particular.

## Introduction

A variety of proteins have the ability to self-assemble and form fibrillar aggregates. Protein aggregation and accumulation of aggregated proteins are responsible for various diseases which have been collectively named as protein conformational disorders (PCDs) [1–5]. PCDs include Parkinson disease, Alzheimer's disease, Huntington disease, haemolytic anaemia, type II diabetes mellitus, cystic fibrosis, dialysis-related amyloidosis and many other diseases [3, 5].The proteins involved in PCD are misfolded compared to their native state, and contain beta sheet rich structures termed as amyloid fibrils [4,5]. In view of the importance of protein fibrillation in various diseases, many efforts have been made to find molecules which can inhibit the fibrillation process [6–11].

Chemical chaperones are small molecules which are known to have positive influence on protein folding and stability [12]. They do so by influencing the rate of the folding reaction and thereby stabilize properly folded states of proteins [13]. Chemical chaperones can be classified into two categories; osmolytes and hydrophobic molecules [14]. Osmolytes are small organic molecules occurring naturally which have the ability to minimize osmotic stress. They stabilize the native state of proteins by raising the free energy of the unfolded state thereby driving the folding equilibrium toward natively folded conformations [15]. Osmolytes mainly include amino acids and their derivatives, polyols, sugars and methylamines. Compounds such as glycine, N-methylglycine, N,N-dimethylglycine, N,N,N-trimethylglycine, trimethyl-N-oxide, polyethylene glycols, sucrose, trehalose, glycerol and many others also fall under this category and have been found to increase stability in several proteins[15–19]. Likewise, many compounds such as glycerol, sorbitol, sucrose, glycine, proline, betaine, sarcosine and trimethyl-N-amine have been found to be effective against protein aggregation [17, 19]. Considering the direct influence of chemical chaperones on proteins’ structure, stability, solubility and folding reactions [19, 20] it may be envisaged that they may modulate the tendency of the proteins to aggregate and also the nature of the aggregates that would be formed [21–24].

We have selected two osmolytes, proline and sorbitol to quantitatively investigate the influence of chemical chaperones on inhibition of protein fibrillation. Proline is a multifunctional amino acid and well known chemical chaperone which is reported to protect citrate synthase against thermo-denaturation, and also stimulates renaturation of citrate synthase after urea denaturation [19]. In addition to the osmo-protection and thermo-protection of citrate synthase, proline has also been demonstrated as anti-aggregation agent of the same [25]. Proline accumulation is associated with stress adaptation such as salt tolerance, high light and UV irradiation, oxidative and biotic stress in many plants including rye grass [26], some rice varieties [27], barley [28], arabidopsis [29], nicotiana [30] and others. Proline, betaine and 4-hydroxyprolinebetaine which are formed by modifications of proline also serve as very good osmoprotectants in E. coli and some other higher plants [31, 32].

Sorbitol is known to improve production of soluble recombinant proteins in E. coli [33] and also increases solubility of intracellular FVIII aggregates, hence improves transport from endoplasmic reticulum to golgi in mouse models [34]. Sorbitol is also reported to be involved in initiation and facilitation of proper folding of several mutants of human cystathionine β-synthase [35]. In *Bacillus licheniformis* it is engaged in refolding of guanidine hydrochloride denatured trehalose-6-phosphatehydrolase [36]. Some simulation studies have suggested that sorbitol modifies the peptide conformational dynamics, and thus alters the aggregation kinetics as well as the physical characteristics of assembling amyloid fibrils [37].

The proteins selected for the present study are hen egg white lysozyme and bovine pancreatic insulin. Hen egg-white lysozyme isa129 amino acids containing enzyme that lyses the cell walls of bacteria and has been extensively studied. Structurally, the monomeric form of the lysozyme has four disulfide bonds and adopts mainly helical conformation (~30% α-helix; ~6% β- sheet) [38, 39]. This protein is closely related to human lysozyme, the variants of which were shown to form amyloid fibrils that were related to hereditary systemic amyloidosis [40, 41]. The other protein, insulin, is 51 amino acids residue long protein hormone which regulates transport of glucose from blood in cells[42]. Fibrillation of insulin reduces its effectiveness and is responsible for injection amyloidosis, observed in insulin dependent diabetic patients who receive insulin frequently [43, 44]. Lysozyme and insulin have tendency to undergo fibrillation process under adverse environmental conditions such as high temperature, low pH, high concentration, and incubation in presence of moderate concentrations of co-solutes/co-solvents [45–47]. These features make the studied proteins suitable models for studying inhibition of the fibrillation process. The present work reports an interesting observation that proline and sorbitol exhibit a synergy in the fibrillation process of the two proteins. It is conceivable that such effects would be observed for many different osmolyte combinations. Such a phenomenon would have important implications for combination therapy with different types of drugs.

## Materials and Methods

The chemicals hen egg white lysozyme (>0.95), bovine pancreatic insulin (>0.95), L-proline (>0.99), sorbitol (>0.95) and thioflavin T (dye content 0.65–0.75) were procured from Sigma-Aldrich Chemical Company USA. The listed purities of these compounds, on mass fraction basis, are given in the parenthesis. All the solutions were prepared in milliQ water from Merk Millipore system. The lysozyme solution was prepared in 40 mM phosphate buffer at pH 7.0 and insulin solution was prepared in 20 mM phosphate buffer at pH 2.0. The stock solutions of each protein were dissolved initially in the respective buffers and dialyzed overnight extensively at 4°C against the buffer with at least three changes of the latter. All other solutions were prepared in the final dialysate buffer. The pH of the solution was measured on a Pico+ pH meter from Lab India Pvt. Ltd. at ambient temperature.

### In-vitro lysozyme and insulin fibrillation

The concentration of the protein was determined on a UV-1800 Shimadzu UV-visible spectrophotometer. The value of extinction coefficients used for lysozyme and insulin were 26.5 at 280 nm [48] and 1.0 at 276 nm [49] for 1.0 mg/ml protein respectively. For preparation of lysozyme fibrils the experimental conditions explained by Vernaglia *et al* have been used [50]. Lysozyme solutions were prepared in 40 mM phosphate buffer (pH 7.0) at a concentration of 140 μM and in presence of 4M guanidine hydrochloride [50]. Where as in case of insulin, the samples were prepared in 20 mM phosphate buffer (pH 2.0) at a concentration of 512 μM [51]. Both proteins were incubated at 37°C and accompanied with stirring at 250 rpm to induce fibrillization [51].

### Thioflavin T fluorescence measurements

Thioflavin T (ThT) is cationic benzothiazole dye, widely used as a marker to detect protein fibrillation because of their tendency to bind cross β sheet structures of amyloid fibrils [52]. All the fluorescence measurements were done on a Agilent spectrofluorimeter with excitation and emission slit widths fixed at 5 nm. A stock solution of ThT was prepared in phosphate buffer (20 mM, pH 2.0). The concentration of ThT was determined by using an extinction coefficient *E* = 26,620 M^1^cm^-1^ at 412 nm using a spectrophotometer [53]. At different time intervals an aliquot of incubated sample solution was mixed with ThT solution such that the final concentrations of protein and ThT for the fluorescence measurements were 5 μM and 50 μM, respectively. The samples were excited at a wavelength 450 nm and emission was detected at 480 nm [54]. The acquired data from ThT fluorescence measurements were fitted to the sigmoid curve represented by the following equation [55]

$$Y=y_{i}+m_{i}x+\frac{y_{f}+m_{f}x}{1+e^{-[(x-x_{0})/\tau ]}}$$ (1)

where *Y* is the fluorescence intensity, *x* is time, and *x*_0_ is the time to reach 50% of maximal fluorescence. Thus, the apparent rate constant, *k*_app_, for the growth of fibrils is given by 1/*τ*, and the lag time is given by *x*_0_−2*τ*. Each experiment was performed at least three times and an average was used.

### Transmission electron microscopy

The protein was diluted to a concentration of 20μM and 10μL aliquot was placed on the Formvar-coated 300 mesh copper grids (Ted Pella) and allowed to adsorb onto the surface of the grid for 5 min. The grid was then washed three times (30 s each time) with filtered water at pH 2.7 and then negative stained with 2% aqueous uranyl acetate for few minutes. After pre-rinsing with large volumes of water and being dried in air, the grids were examined to acquire images on a JEOL JEM-100B Transmission Electron Microscope which operates at an accelerating voltage of 80 kV.

### Isothermal Titration Calorimetry

The interaction of the insulin amyloid fibrils with the individual osmolytes and the mixture of osmolytes was studied by using a VP isothermal titration calorimeter (Microcal LLC Northampton MA). Experiments were carried out by titrating fibril solution into the sample cell containing buffer or appropriate amount of the osmolyte in aliquots using a rotating stirrer-syringe of 250 μl capacity. The reference cell was filled with the respective buffer. The experiments were designed for a total of 10 consecutive injections, each having a volume of 10 μl of 0.517mM native insulin solution or heat induced fibril solution into buffer or osmolyte solution in the cell. The duration between consecutive injections was 10 s with an interval of 4 min between each injection. The same procedure was used to measure the heats of dilutions by titrating buffer with the respective osmolytes at the same concentrations as used in the main experiments. After dilution corrections, the ITC profiles were analyzed to determine the heat of interaction by using Origin 7.0 software supplied by Microcal.

## Results and Discussion

### Insulin and lysozyme fibril formation

**Fig 1:**
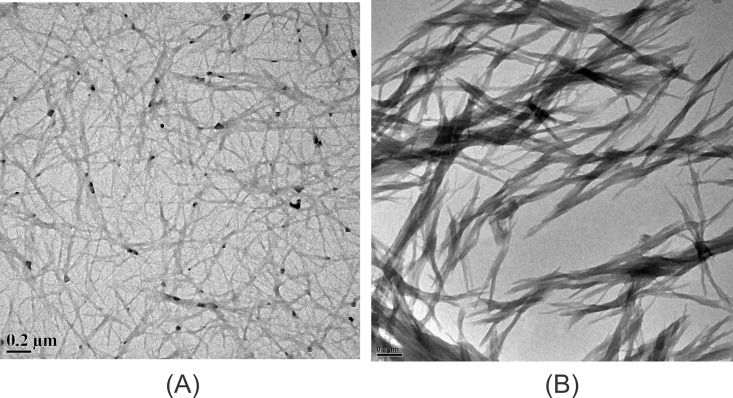
Transmission electron microscopic (TEM) images of (A) insulin and (B) lysozyme fibrils after 36 h of incubation.

In order to do kinetics studies for fibrillation, bovine pancreatic insulin and hen egg white lysozyme were incubated at 37°C with stirring rate of 250 rpm. Bovine pancreatic insulin and hen egg white lysozyme solutions were prepared in 20 mM phosphate buffer at pH 2.0 and 4M guanidium hydrochloride at pH 7.4 respectively. The control experiments were done to check the effects of pH and GuHCl on ThT fluorescence (S1A and S1B Fig). The fibril extension was monitored using ThT which interacts mainly with amyloid fibrils and gives characteristic emission maximum at 482 nm when excited at 450 nm[54]. The fibrillation curves for both proteins show sigmoidal behaviour consisting of three distinct phases; initial lag phase, a subsequent elongation phase and a final saturation phase (S2A and S2B Fig). The lag time for insulin and lysozyme fibrillation was calculated using Eq 1 and is found to be (12.7±0.1) h and (1.5±0.3) h with growth rate constants of (1.32±0.06) h^-1^ and (0.98±0.14) h^-1^, respectively. In order to further confirm the insulin and lysozyme fibrillation independently, transmission electron microscopy (TEM) was performed. Fig 1A and 1B present the TEM images of insulin and lysozyme fibrils taken after 36 h of incubation.

### Effects of proline and sorbitol on insulin and lysozyme fibrillation

**Fig 2:**
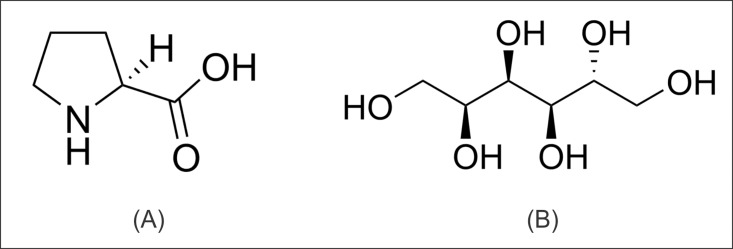
Chemical structures of osmolytes (A) proline and (B) sorbitol.

We have studied here the effects of proline and sorbitol on insulin and lysozyme fibrillation. Proline and sorbitol are small molecules having different features with respect to polarity, charge, and H-bonding abilities, which could result in different types of interactions with the protein surface. Proline contains a closed ring structure in its side chain (Fig 2A) which has a hydrophobic surface and thus would interact with proteins via hydrophobic interactions. On the other hand sorbitol possesses multiple hydroxyl groups (Fig 2B) which can participate in formation of hydrogen bonds with side chains of proteins.

**Fig 3:**
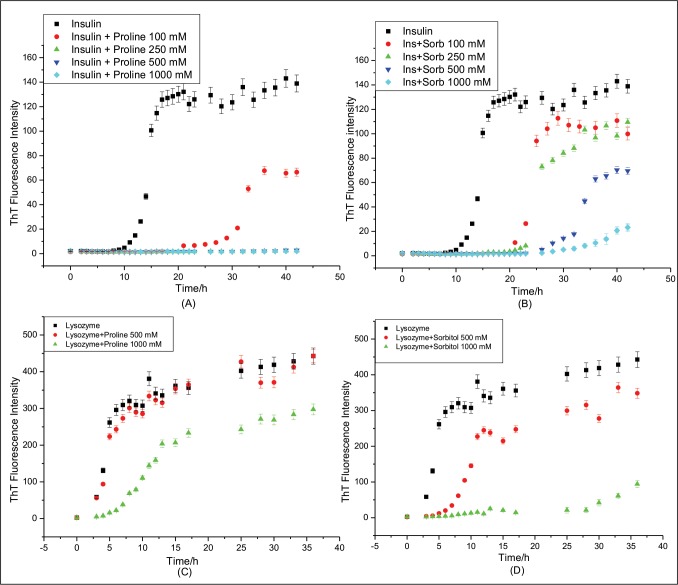
Kinetics of insulin (A) and (B) and lysozyme (C) and (D) fibril extension in absence and presence of different concentration of proline and sorbitol respectively.

Fig 3A presents the time course of insulin fibrillation in absence and presence of different concentrations of proline. It is evident from the ThT kinetics plot that the lag period has significantly increased in presence of 100 mM proline (Table 1). Further, increase in the concentration of proline has completely suppressed the fibrillation of insulin. When the ThT kinetics studies were performed in presence of increasing concentrations of sorbitol from 250 mM to 1000 mM, it showed inhibition of insulin fibrillation in a concentration dependent manner (Fig 3B). It is clear from the figure that sorbitol has not only delayed the fibril formation but has also decreased the amount of fibrils formed.

**Table 1:** Fibrillation kinetics parameter for Insulin and lysozyme.

System	Lag time (t)/h	Growth rate constant (*k*app)	Amplitude
Insulin	12.7±0.1	1.32±0.06	127±7
Insulin+Proline (100 mM)	29.4±0.1	0.84±0.24	65±2
Insulin+Sorbitol (100 mM)	22.4±0.2	1.51±0.11	105±10
Insulin+Proline+Sorbitol (100 mM)	27.3±0.1	0.39±0.02	53±2
Lysozyme	1.5±0.3	0.98±0.14	370±27
Lysozyme+Proline (500 mM)	2.1±0.2	0.86±0.07	344±25
Lysozyme+Sorbitol (500 mM)	6.8±0.04	0.93±0.15	294±11
Lysozyme+Proline+Sorbitol (500 mM)	15.1±0.4	0.28±0.06	252±6

Fibrillation kinetics parameter for 512 μM bovine pancreatic insulin and 140 μM hen egg white lysozyme in absence and presence of proline, sorbitol and their mixture.

Similarly, when the ThT fluorescence kinetics studies for lysozyme fibrillation were performed in absence and presence of 500 mM proline (Fig 3C), we could not find appreciable change either in the lag period or in the amount of fibrils formed (Table 1). Effect of lower concentration of proline (100 mM) on lysozyme fibrillation was also studied (data not shown) and was not found effective. Nonetheless, there is a considerable delay in the lag period as well as decrease in the ThT fluorescence intensity when the concentration of proline was increased to 1000 mM (Fig 3C). From these observations it is clear that proline acts as inhibitor of protein fibrillation for both proteins but it is more effective in case of insulin compared to lysozyme even at lower concentrations. The kinetics of lysozyme fibrillation was also studied in presence of different concentrations of sorbitol (Fig 3D). Here also, similar to its effect on insulin fibrillation (Fig 3B), concentration dependent inhibition of lysozyme fibrillation was observed.

**Fig 4:**
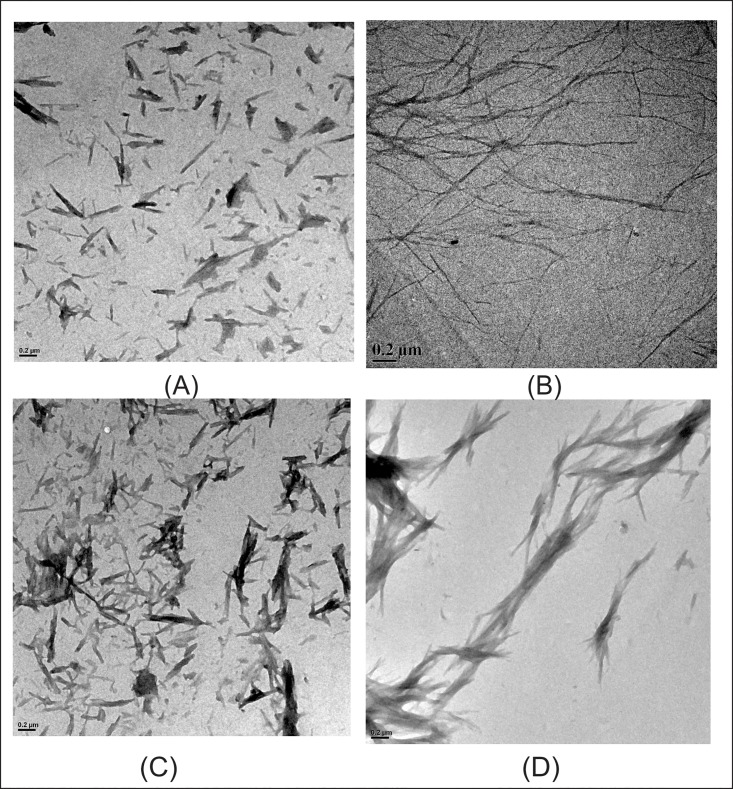
TEM images of insulin (A) and (B) in presence of 100 mM proline and sorbitol respectively and lysozyme (C) and (D) in presence of 500 mM proline and sorbitol respectively.

Fig 4A and 4B present TEM images of insulin in presence of 100 mM proline and 100 mM sorbitol, respectively, taken after 36 h of incubation. It is clear from the figure (Fig 4A) that in presence of 100 mM proline, insulin forms much smaller and thicker fibrils which have completely altered morphology. In contrast, insulin fibrils formed in presence of 100 mM sorbitol are only slightly different from those formed in its absence (Fig 4B). TEM image of lysozyme taken in presence of 500 mM proline shows bundle like fibrils (Fig 4C) and are much smaller compared to those taken in absence of proline (Fig 1B). Here in presence of 500 mM proline, the extent of fibrillation is similar to that in the absence of 500 mM proline but the morphology of fibrils has changed. That means, instead of forming longer fibrils shorter fibrils have formed. Fig 4D presents TEM image of lysozyme when incubated in presence of 500 mM sorbitol. Here the morphology of lysozyme fibrils in presence of 500 mm sorbitol has not changed significantly (Fig 4D) but the amount of fibrils has significantly decreased compared to that in the absence of sorbitol (compare Figs 1B and 4D). Thus the effects of the two osmolytes with regard to morphology and extent of fibrillation are different.

### Synergistic inhibition of protein fibrillation by proline and sorbitol

We carried out ThT binding assays for insulin and lysozyme fibrillation in presence of mixture of proline and sorbitol. Here the main objective is to see the effect of one osmolyte on protein aggregation in presence of another osmolyte and vice-versa. This can, in principle, be done in two ways: (i) reducing the concentration of each of the osmolytes to half in the mixture compared to that when present alone so that the total osmolyte concentration is maintained and (ii) keeping the concentration of each osmolyte the same in the mixture as when they are present individually. In the former case, the extent of interaction of perturbation of osmolyte with the protein is reduced by virtue of reduction in the number of molecules. In that situation it is difficult to assess whether the observed effect is due to the presence of the second osmolyte or due to reduced interaction. Therefore, in order to be able to assess the effects of one osmolyte on the interaction of another osmolyte with the protein quantitatively when both are present, it is important to keep the concentrations the same as in the individual cases.

**Fig 5:**
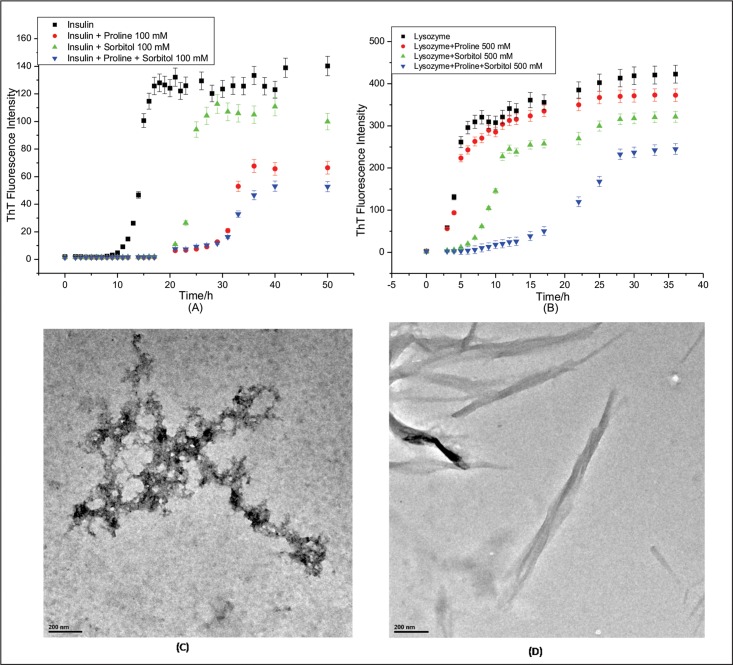
Kinetics of (A) insulin and (B) lysozyme fibrillation monitored by the binding of ThT with amyloid fibrils in presence of proline, sorbitol and their mixture, and TEM images of (C) insulin and (D) lysozyme in presence of mixture of proline and sorbitol.

Fig 5A presents kinetics of insulin fibrillation in absence and presence of 100 mM proline and sorbitol and in their mixture (100 mM each).The lag period of insulin fibrillation in presence of proline-sorbitol mixture is considerably more (27.3±0.1) h than in the presence of 100 mM sorbitol (22.4±0.2) h and is comparable to that in the presence of 100 mM proline (29.4±0.3) h (See Table 1). The apparent rate constants (*k*_aap_) for insulin fibrillation in presence of 100 mM proline, 100 mM sorbitol and in their mixture are (0.84±0.24) h^-1^, (1.51±0.11) h^-1^and (0.39±0.02) h^-1^, respectively (Table 1). Interestingly, even though the apparent rate constant increases in presence of sorbitol as compared to that in protein alone, the value comes down substantially in the presence of the mixture. These values clearly demonstrate that the mixture of 100 mM proline and sorbitol is effective not only in delaying the lag period but also in controlling the apparent rate constant (*k*_aap_) of insulin fibrillation process. The maximum ThT intensity obtained in case of proline-sorbitol mixture is also less compared to that from the individual components. TEM images of insulin (Fig 5C) taken in presence of mixture of proline and sorbitol also supports ThT kinetics data. The morphology of insulin fibrils has entirely changed in presence of mixture and shows resemblance to amorphous aggregates.

The results of ThT binding studies for lysozyme aggregation in presence of 500 mM proline, sorbitol and their mixture are shown in Fig 5B. The lag time for lysozyme fibrillation in presence of proline and sorbitol mixture was (15.1±0.4) h, which is significantly delayed than in presence of proline or sorbitol alone [(2.1±0.2)h and (6.8±0.3)h respectively]. Here also the mixture of proline and sorbitol (250 mM each) has significantly reduced the apparent rate constant (*k*_aap_) of lysozyme fibrillation [(0.28±0.06)h^-1^] compared to that when proline and sorbitol are present individually (Table 1). The TEM image of lysozyme taken after 36 h of incubation in presence of 500 mM proline and sorbitol mixture shows short and thick lysozyme fibrils. All these observations put together indicate an interesting synergy between the effects of proline and sorbitol in the inhibition of fibrillation of the two proteins.

### Thermodynamics of fibrillation

On the basis of ThT kinetics experiments it is clear that the two proteins behave and interact differently with the individual osmolytes and their mixture. Proline was found to be more effective compared to sorbitol in case of insulin whereas the results were reverse in the case of lysozyme. In addition, the two proteins show different behaviors in the mixture of osmolytes. This difference must be attributed to different external surfaces of the two proteins which are involved in various interactions. In general, the interaction and consequent synergy would depend on the surface exposed hydrophobic residues and electrostatic potential on the protein surface. It is well established that the interaction between molecules at large distances is determined almost exclusively by the electrostatic potentials [56], and therefore the interaction behavior of proline and sorbitol with the protein would be expected to be dependent on the potential surface of the protein. Previous studies suggest that upper and lower surfaces of oligomeric bovine insulin have greater hydrophobicity than other small globular proteins [57]. In contrast, a fraction of lysozyme surface is either free from hydrophobic regions or bears some electrostatic potential near hydrophobic patches [58].

In the light of this background it is very important to identify the modes of interaction between the proteins and the osmolytes at different stages of fibrillation. To understand the mechanism of inhibition of insulin and lysozyme fibrillation by proline and sorbitol and their synergistic effects, isothermal titration calorimetric experiments were performed.

Isothermal titration calorimetry (ITC) is an ultrasensitive technique for thermodynamic characterization of intermolecular interactions. ITC measures the heat released (exothermic reaction) or absorbed (endothermic reaction) by the stepwise addition of ligand to the solution containing the molecule under study. For a binding reaction at equilibrium, standard enthalpic and entropic changes (ΔH° and ΔS° respectively) contribute to the Gibbs free energy of binding (ΔG°) and are expressed by following equation:

$$\Delta G^{o}=\Delta H^{o}-T\Delta S^{o}$$ (2)

Where T is temperature in Kelvin. For spontaneous binding of a ligand to a protein, change in standard Gibbs free energy of the system should be negative. The thermodynamic quantities ΔH° and ΔS° are the main driving forces for protein ligand binding as they determine the sign and magnitude of ΔG°. While ΔH° represents direct heat change, ΔS° represents change in order/disorder in the system and TΔS° contributes to the energy change.

The binding enthalpy (ΔH°) reflects the change in enthalpy resulting from the formation of various noncovalent interactions such as hydrogen bonds, electrostatic interactions, vanderWaals interactions and hydrophobic interactions at the binding interface. The enthalpy change is a global property and a result of formation and disruption of many individual interactions [59] which includes disruption of old solute-solute, solute-solvent interactions and formation of new bonds between protein and ligand and the solvent reorganization around the interacting partners. Thus the observed enthalpy of interaction is exothermic or endothermic depending upon the predominance of specific type of interactions in the interacting system.

In ionic interactions, charged groups of ligand and charged amino acids (histidine, arginine, lysine, aspartate and glutamate) in the binding cavity are involved. These interactions are exothermic in nature. For example, the protonation of imidazole group in histidine contributes approximately -6 kJ mol^-1^ of favourable enthalpy [60]. Similarly protonation of carboxylic group also contributes favourably to the change in enthalpy [61]. There are many reports available which consider ionic interactions in protein-ligand binding and in every case the enthalpy contribution to free energy change is favourable (ΔH° negative) [62–67].

When protein-ligand binding takes place in aqueous medium, various hydrogen bonds are formed such as amide-water hydrogen bond, polar side chains-water hydrogen bond, side chain-ligand hydrogen bond etc [68, 69]. It has been found that ΔH° for hydrogen bond is between -8 to -13 kJmol^-1^. Another type of interactions for protein-ligand binding is hydrophobic interactions which is mainly accompanied by disruption of energetically favourable non-covalent interactions. This involves major structural reorganization of the solvent water molecules leading to hydrophobic association of the protein-ligand surfaces [70]. More explicitly, there will be (i) breaking of ordered H-bonds among the water molecules on the hydrophobic surface of the protein, (ii) transfer of the ordered water molecules to the less ordered state in bulk water and (iii) association of the hydrohobic surfaces of the protein and the ligand via van der Waal’s interactions [70]. The first part produces positive enthalpic change (ΔH°), the second part produces heat due to positive entropic change (TΔS°) and the third also results in positive enthalpic change [70]. In atypical ITC measurement, ΔH° and ΔS° are separately measured. For a protein-ligand complex the net values and sign of the enthalpy change (ΔH°) are a result of combination of different types of interactions involved in the binding process.

**Fig 6:**
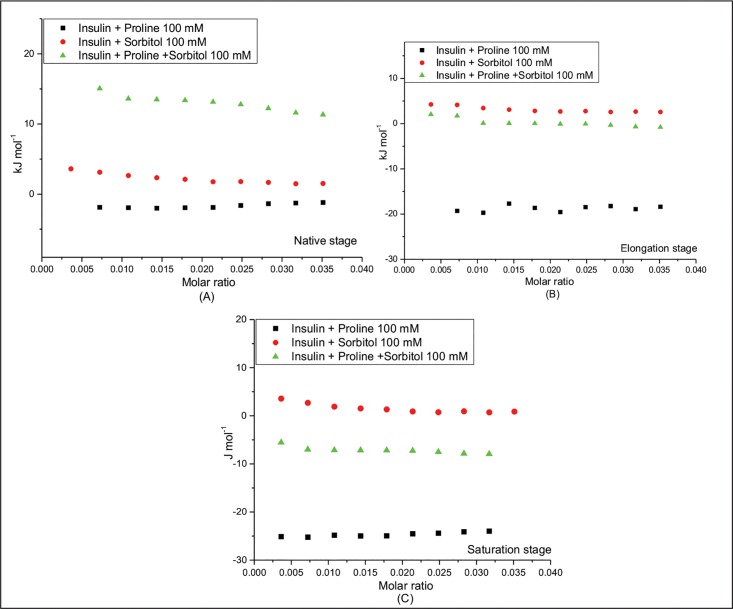
Enthalpies of interaction of insulin in presence of 100 mM proline, sorbitol and their mixture at (A) native, (B) elongation and (C) saturation stages of fibrillation.

**Fig 7:**
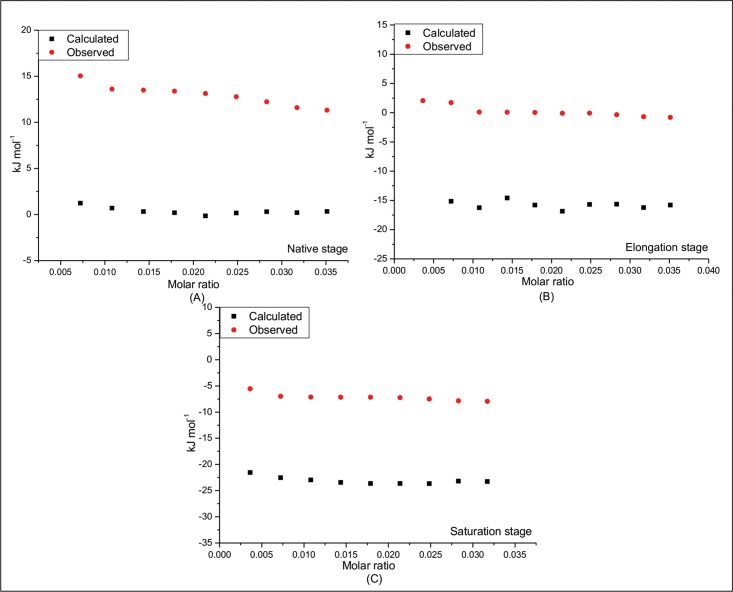
Comparison between sum of the enthalpies of interaction of insulin in presence of 100 mM proline and sorbitol individually and in their mixture at (A) native, (B) elongation and (C) saturation stages of fibrillation.

In the present case the ITC experiments were performed with native proteins (insulin and lysozyme) and with the proteins at different stages of fibrillation, in presence of proline, sorbitol and their mixture. It is understood that the extent of inhibiton of protein fibrillation will depend on the interactions involved between the protein andthe inhibitor, which in turn is determined by their molecular properties. In all the ITC experiments, proline/sorbitol solutions were taken in the sample cell and the protein solution in native or different stages of fibrillation was taken in the syringe. Figs 6 and 7 present the ITC profiles accompanying the interaction of insulin and lysozyme under these conditions, respectively. It is seen that the interaction of the protein (insulin or lysozyme) with the osmolyte does not follow any typical binding profile leading to saturation levels. These observations suggest that neither lysozyme nor insulin has specific binding site for proline or sorbitol. In such a situation we can only extract ΔH from the data. It turns out that the interaction of these proteins with the osmolytes is exothermic in some cases and endothermic in some others.

The interaction of native insulin with 100 mM proline is observed to be exothermic with enthalpy values changing from -(1.89±0.20) kJmol^-1^ to -(1.20±0.10) kJmol^-1^during the course of 10 injections. However, the heat of interaction turns endothermic with 100 mM sorbitol with the enthalpy values changing from (3.60±0.03) kJmol^-1^ to (1.53±0.02) kJmol^-1^. Interestingly, when native insulin is titrated with the mixture of proline and sorbitol each at 100 mM concentration the heat of interaction becomes further endothermic with enthalpy values in the range of (15.04±0.20) kJmol^-1^ to (11.20±0.10) kJmol^-1^.

Fig 6B presents the interaction of insulin at elongation stage. Here also the interaction with proline is exothermic but is endothermic with sorbitol or the mixture of the two. These results suggest that proline is able to establish polar interactions with the protein more at the elongation stage [ΔH values ranging from -(19.31±0.20) kJmol^-1^ to -(18.38±0.10) kJmol^-1^] than with the native protein. The relationship of polar interactions with the protein is established based on the basis of observed exothermic effects which are more than that with the native state. But in spite of large number of hydroxyl groups present, sorbitol is not able to establish such interactions with the protein at the elongation stage.

Similar trend is observed in the interaction of the two osmolytes with mature fibrils. The extent of polar interaction with proline is highest as seen from the exothermicity in the mixture [ΔH values ranging from -(25.11±0.03) kJmol^-1^ to -(23.97±0.03) kJmol^-1^]. On the other hand, sorbitol interacts with protein fibrils in an endothermic manner, though in the mixture, the interaction becomes exothermic.

It was seen from ThT fluorescence data (Fig 5A) that proline and mixture of proline and sorbitol inhibit the fibrillation more strongly than sorbitol alone. This prevention appears to be facilitated by establishment of polar interactions by the osmolyte with insulin at the elongation and even with the mature fibrils. This observation is supported by the ITC results in which the enthalpy of interaction of insulin with proline becomes more exothermic as the fibrillation proceeds.

ITC data of lysozyme could not be analysed because of high heat of dilution of guanidium hydrochloride which masks actual heat of interaction of lysozyme with the osmolytes. The extent of synergy in case of insulin aggregation can be understood by comparing the experimental value of enthalpy of interactions in mixture of osmolytes with that of the sum total of the individual enthalpy values. Fig 7 presents a comparison between the sum and the observed values of enthalpy of interactions at different stages of fibrillation. The calculated values of enthalpy of interaction are slightly endothermic [(1.23±0.03) to (0.32±0.01) kJmol^-1^] for native stage and becomes more and more exothermic at elongation [-(15.14±0.23) to -(15.79±0.09) kJmol^-1^] and saturation stages [-(21.53±0.06) to -(23.25±0.08) kJmol^-1^] respectively. The observed values of enthalpy of interaction are less exothermic compared to those of calculated values at all stages of fibrillation. This decrease in exothermicity can be a consequence of involvement of hydrophobic interactions which contribute towards endothermicity. It is well known that hydrophobic interactions are the main driving force for aggregation. Thus, it is clear from the above data that the mixture of osmolytes has ability to interfere in both electrostatic as well as hydrophobic interactions that may be causing protein self-association leading to fibrillation and the observed synergy between the osmolytes is an outcome of a co-operative effect of one osmolyte on the other, with regard to their interactions with the protein.

**Fig 8:**
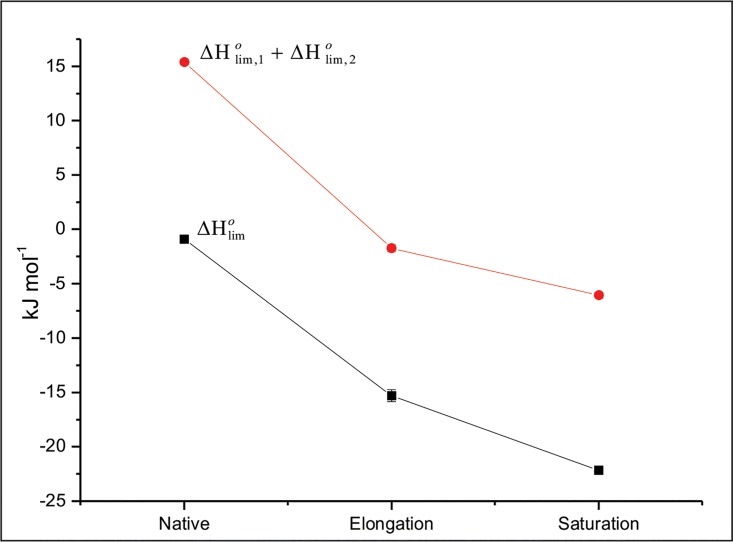
The sum of limiting standard enthalpies of interaction of insulin in 100 mM proline and sorbitol individually and in the mixture of osmolytes at different stages of fibrillation.

We have also calculated here the limiting standard enthalpy of interaction ($\Delta H_{\lim}^{o}$), which is indicative of the nature of solute-solvent interactions without contributions from solute-solute and solvent-solvent interactions. In all the above cases the heat of interaction was observed to be concentration dependent, and hence the value of $\Delta H_{\lim}^{o}$ was obtained from a linear fit to the experimental data points. Fig 8 presents the values of limiting standard enthalpy of interactions ($\Delta H_{\lim}^{o}$) of insulin with proline and sorbitol for the sum total of individual osmolytes and observed enthalpies in the mixture of two osmolytes at native, elongation and saturation stages of fibrillation of insulin. The extent of synergy can be correlated with excess enthalpy ($\Delta H_{\lim}^{o}{}^{\left(Excess\right)}$) and can be calculated using the following equations;

$$OS_{1}+P\overset{\Delta H_{\lim,1}^{o}}{\to }OS_{1}P$$ (3)

$$OS_{2}+P\overset{\Delta H_{\lim,2}^{o}}{\to }OS_{2}P$$ (4)

where *OS*_1_, *OS*_2_, and *P* represent first osmolyte, second osmolyte and protein respectively. $\Delta H_{\lim,1}^{o}$ and $\Delta H_{\lim,2}^{o}$ are limiting enthalpies per mole of protein.

$$OS_{1}+P+OS_{2}\overset{\Delta H_{\lim}^{o}}{\to }OS_{1}OS_{2}P$$ (5)

Ideally, if there is no synergy, ΔH^*o*^ should be sum of $\Delta H_{\lim,1}^{o}$ and $\Delta H_{\lim,2}^{o}$ which can be represented as

$$\Delta H_{\lim,1}^{o}+\Delta H_{\lim,2}^{o}=\Delta H_{\lim}^{o}$$ (6)

However, if synergy occurs, the excess enthalpy $\Delta H_{\lim}^{o}{}^{\left(Excess\right)}$ will be an indicator of the extent of synergy represented by the following equation.

$$\Delta H_{\lim}^{o}{}^{\left(Excess\right)}=\Delta H_{\lim}^{o}-\left(\Delta H_{\lim,1}^{o}+\Delta H_{\lim,2}^{o}\right)$$ (7)

Table 2 summarizes the values of $\Delta H_{\lim}^{o}{}^{\left(Excess\right)}$ for insulin in mixture of proline and sorbitol at different stages of fibrillation. The limiting standard enthalpies of interaction ($\Delta H_{\lim,1}^{ο}$ and $\Delta H_{\lim,2}^{ο}$) for sum of the individual osmolytes at native, elongation and saturation stages are -(0.89 ± 0.28), -(15.30±0.56) and -(22.19±0.38) kJmol^-1^ respectively. The observed values of the limiting standard enthalpies of interaction in the mixture of osmolytes ($\Delta H_{\lim}^{o}$) are (15.40±0.27), -(1.74±0.32) and -(6.06±0.28) kJmol^-1^for native, elongation and saturation stages, respectively. The observed value of the limiting standard enthalpies of interaction in the mixture of osmolytes ($\Delta H_{\lim}^{o}$) is less exothermic compared to that of calculated values. The values of excess enthalpy ($\Delta H_{\lim}^{o}{}^{\left(Excess\right)}$) for native, elongation and saturation stages are 16.29, 13.56 and 16.13 kJmol^-1^ respectively. The values of $\Delta H_{\lim}^{o}{}^{\left(Excess\right)}$ represents a quantification of synergy in terms of energetics and indicates modified interactions between insulin and mixture of osmolytes compared to the individuals.

**Table 2:** The limiting standard enthalpies of interaction ($\Delta H_{\lim}^{o}$).

Stage	ΔH°_*lim*_ (Calculated)/kJmol^-1^	ΔH°_*lim*_ (Calculated)/ kJmol^-1^	ΔH°_*lim*_^*(Excess)*^/ kJmol^-1^
Native	-(0.89±0.28)	(15.40±0.27)	16.29
Elongation	-(15.30±0.56)	-(1.74±0.32)	13.56
Saturation	-(22.19±0.38)	-(6.06±0.28)	16.13

The limiting standard enthalpies of interaction ($\Delta H_{\lim}^{o}$) of 512 μM bovine pancreatic insulin with 100 mM proline, sorbitol and their mixture at different stages of fibrillation.

All the above data on energetics of interactions derived from ITC suggest that the osmolytes reduce the intermolecular electrostatic as well as hydrophobic interactions in proteins resulting in inhibition of protein fibrillation.

## Conclusions

Insulin and lysozyme form fibrils at 37°C under partial denaturing conditions. ThT binding assay and TEM confirm the onset of fibrillation in bovine pancreatic insulin and hen egg white lysozyme after an initial lag phase of (14.2±0.2) h and (1.5±0.1) h, respectively. Proline and sorbitol suppress the fibrillation of proteins, and the action of the osmolytes is synergistic. The values of limiting standard enthalpies of interaction of osmolytes and insulin obtained from ITC suggest prevention of β-sheet stacking and assembly by establishing polar and hydrophobic interactions between the osmolytes and the protein chains. The interaction of osmolytes with the proteins is observed to be protein specific. The synergy observed between the two chosen osmolytes can be representative and can be expected to happen with many others. This has implications for combination therapy against protein fibrillation and diseases, in general.

## Supporting Information

S1 Fig Control experiments. Control experiments showing the fluorescence properties of ThT in absence and presence of proline, sorbitol and their mixture in (A) buffer at pH 2.0 and (B) buffer containing 4M GuHCl at pH 7.0.
(TIF)

S2 Fig ThT binding assay. Kinetics of (A) bovine pancreatic insulin, (B) hen egg white lysozyme fibrillation monitored by the binding of ThT with amyloid fibrils.
(TIF)
